# Against ‘immigrant integration’: for an end to neocolonial knowledge production

**DOI:** 10.1186/s40878-018-0095-1

**Published:** 2018-09-25

**Authors:** Willem Schinkel

**Affiliations:** Erasmus University Rotterdam, Rotterdam, Netherlands

**Keywords:** Immigrant integration, Race, Colonialism, Social science, Multiculturalism

## Abstract

This paper, written on invitation by the editors of Comparative Migration Studies, is intended as a provocation piece for invited commentators, and more broadly for those working with, or concerned about, the field of immigrant integration research. It outlines an argument put forward in Imagined Societies. A Critique of Immigrant Integration in Western Europe (Cambridge University Press, 2017) that 1) critiques immigrant integration research for bad (or lacking) conceptual work, specifically also in regard to the core sociological notion of ‘society’; 2) argues that immigrant integration monitoring is a neocolonial form of knowledge intricately bound up with the contemporary workings of power, and 3) proposes social science moves beyond notions of ‘immigrant integration’ and ‘society’ towards an imagination against the grain that involves paying due attention to what happens when migrants move across social ecologies, without resorting to commonsense and/or policy categories in doing so.

## Introduction: Waking up from ‘multiculturealism’

There is no denying that today, researching migration and immigrant integration in Western Europe occurs amidst a public discourse that is highly toxic. It is toxic because it is riddled with racisms hard to avoid when, as a researcher, one wishes to conduct research in the established way, i.e., when one wishes to acquire research funds and publish in high-ranking journals about ‘immigrant integration’. In general, immigrant integration research occurs under the sign of a putatively ‘failed multiculturalism’. This fiction of a multiculturalism that was once dominant across Western Europe but has now been shown to have failed is espoused both by politicians of nearly all colors and by well-known immigrant integration scholars (cf. Koopmans, 2013). But this multiculturalism of the past is hardly ever sketched in detail, as indeed it cannot, because it has never been implemented across Western Europe. When government leaders such as Merkel, Sarkozy and Cameron gave their by now famous multiculturalism-denouncement speeches, the astute observer should have concluded right away that these breaks from a multiculturalist past were indeed rhetorical breaks, which conjured a fictive past in order to change the present. For the UK, Germany and France have never had a shared policy on immigrant integration. They did not even share the same language with which to describe what is now increasingly commensurated as ‘immigrant integration’. France is perhaps the clearest case of a country that has never had a ‘multiculturalism’ in policy nomenclature, let alone in practice. The same goes for countries such as Belgium, Norway, Sweden, Denmark and the Netherlands – each has had its explicit denouncements of ‘multiculturalism’, but each never had a policy remotely resembling something that might seriously be called that way. In the Netherlands, for instance, ‘multiculturalism’ became a concept frequently used *after* it supposedly was a policy, namely in critiques debunking that supposed but non-existent policy.

In my book *Imagined societies*. *A Critique of Immigrant Integration in Western Europe* (2017), I take this discursive paradox of starting to talk about ‘multiculturalism’ no sooner than the moment one claims to depart from it, as a starting point to discuss the pitfalls of ‘immigrant integration’ both as a political way to describe the process in which migrants settle, and as a concept in social science to analyze such processes. In this paper, intended for a discussion that takes off from the book, I sketch some the main problems of the way the social sciences have taken up the concept of immigrant integration. I’ll therefore not be concerned with alternative ways of describing the settlement and accommodation of migrants here. Yet the two are of course related. The denouncements of a multiculturalism that never was amount to a discursive strategy I have called *multiculturealism*: the self-declared ‘realism’ of supposedly having been ‘multicultural’ and hence ‘politically correct’, naively ‘left-wing’, ‘ignoring the problems’ (with immigrants, with ‘Islam’, and so on), but of now having become realist, daring the speak the harsh truth about the troubled realities of a failing model of immigrant integration. The discourse of multiculturealism has entailed a license to *problematize* migrant others, i.e., to forego the relational aspects of migration and to focus solely on the position and problems of immigrants and their children, many of whom actually born on European soil. The social science of immigrant integration plays a crucial role in this problematization of migrant others. It provides, in a way, the ‘factual architecture’ within which such problematizations take shape. Yet multiculturealism at the same time imposes severe discursive limits on research. Research, increasingly, becomes part and parcel of the problematization of immigrants and their children, and hence it becomes intricately tied to racist discourses and practices. In this paper, I outline the main reasons for this assessment. I discuss the conceptual problems of immigrant integration research, and I illustrate how the problems emerge in the ways immigrant integration tends to be measured. As in the book, my main case will be the Netherlands, which, though peculiar in specific ways, serves as a prism for certain mechanisms of opposing ‘society’ to ‘immigrants’ that are prevalent in various countries in Western Europe.

## ‘Immigrant integration’: The conceptual quagmire

‘Integration’ is of course a well-known sociological concept that figures prominently in the work of theorists such as Durkheim, Parsons and Habermas. From the Latin *integer*, it connotes an ‘unscathed whole’. And indeed in the work of said theorists, ‘integration’ has reference to a social whole. The larger history of the concept lies in an organicist tradition in which the social was conceived as an integrated body. Integration, in that sense, had to do with the internal adjustment of the parts of a whole, but it was ultimately a property of the whole. As I discuss below, there many problems with such a part/whole conception, according to which there are ‘societies’ that consist of ‘members’. But even if one were to subscribe to such a dualism of organicist origin, one still wouldn’t end up with the theoretically impoverished conception of integration that underwrites conventional immigrant integration research, and that currently still operates as the primary way in which Western European nation-states can imagine the immigrant populations in their midst (Favell, 2003; Korteweg, 2017). In that research, integration ceases to be a property of a social whole, and becomes *individualized* by turning into a property of individual people, such as migrants, their children, unemployed persons, or convicted criminals. In other words, in conventional integration research, individuals can be integrated in various degrees. But this individualization of integration is entirely without theoretical underpinnings. Instead, it rests on commonsensical notions of ‘society’ and its individual ‘members’, and on the historically particular plausibility of the individualist (neo)liberal assumptions of this society as consisting of individual members to whom any ‘misfit’ between the two can be one-sidedly attributed. ‘Integration’ thus changes from a *system state* to a *state of being* of an individual. Lack of immigrant integration thus turns out to have to do with the being of immigrants, and the resulting picture of course ends up pitting ‘society’ over against individuals that are racialized in particular ways, because in order for their being to affect their integration, that being must be somehow problematic.

That the application of ‘integration’ to the level of individuals is in fact rather weird becomes apparent when the antonym of ‘being integrated’ is considered. For the opposite of ‘integrated’ is, of course, ‘disintegrated’. And one can say of a whole that it can be integrated or disintegrated, but obviously one cannot consider an individual as ‘disintegrated’, unless of course one considers the individual as a biological whole, a body that is disintegrated. That individuals cannot be socially distintegrated should signal that they can neither be integrated, i.e., that ‘integration’ is not a description of individual states of being. And yet precisely this strange conceptual concoction is at the very heart of immigrant integration research, in which ‘integration’ in fact goes without antonym and is now *internally* partitioned in degrees of integration. One can thus be ‘well integrated’ or ‘less integrated’ than someone else. This internal partition is thus charged with the task of providing conceptual clarity to a concept without antonym, which thus has a purely internally propped up semantic plausibility. But as a result the concept amounts, in fact, to little more than a floating signifier that works well first and foremost because it translates easily across academia and policy, popular discourse and common sense descriptions, i.e., precisely because it lacks all the friction that a theorized, complex concept gives rise to when it travels across communities of practice. The effect is, of course, that ‘integration’ becomes a decidedly un-social and non-relational concept, which posits a static object (‘society’) over against individuals whose being signifies a certain degree of ‘integration’ as an individual-level trait.

Of course individual-level measurements of immigrant integration are usually immediately aggregated into group-level data. After both conceptualizing and measuring ‘integration’ as an individual state of being, this then constitutes a jump in scales that is, again, wholly without theoretical underpinnings and that constructs ‘groups’ out of mere ascribed statistical population categories. The end result is a combination of individualization and de-individualization. ‘Integration’ is the state of an individual, and hence – in line with the neoliberalization of migration and integration policies (Van Houdt, Suvarierol & Schinkel, 2011) – ‘integration’ becomes a matter of ‘individual responsibility’. But at the same time it is *explained* by means of a de-individualizing maneuver, which clusters people in various states of integration in ‘ethnic groups’, and this chimes well with the communitarianism that Western-European neoliberal citizenship policies are often combined with (Schinkel & Van Houdt, 2010). So if an individual is lacking in ‘integration’, the individualized responsibility for this is at once extended to all members of the ‘group’ to which that individual is considered to belong (again, ‘group’ here means nothing more than an aggregation of attributed ‘ethnic identity’). Lack of integration, so to speak, works infectious, but the very reason for aggregating at group level is, of course, that such aggregation is meaningful in an explanatory sense. Otherwise, why not study all members of a population without separating them into statistically constructed ‘ethnic groups’? And ‘ethnicity’, which of course stands in for ‘race’, is in fact the *only* explanatory element in what is otherwise an exercise of description, of classification and of monitoring. No economic factors can emerge in any explanatory way, for instance, because they are *part of* the variables (in the form of SES scores, for instance) that *define* ‘immigrant integration’. They are thus, by design, excluded from any explanatory role in the differentiation between degrees of ‘integration’ that is measured.

So what we end up with is a concept, and a practice, that is thoroughly purified both from notions of class and of race. And such purification can be said to be part of the social ‘function’ of the measurement of immigrant integration, that is, it is part of the contingent way in which ‘immigrant integration’ sustains a classed and raced form of dominance that is less precisely called ‘native’ or even ‘nativist’ than ‘white’. In order to understand this, one must consider what I have discussed in the book as *dispensation of integration*. This is what is ‘granted’, so to speak, to white citizens. And this is the ‘positive’ way of describing the fact that these *do not appear on the integration monitor*. It is the active way to describe an omission that is consequential, and which *already does all the work of separating* those who are considered to make up ‘society’ and those who do not and who thus need to further ‘integrate’. Dispensation of integration means that white citizens are not researched or described in terms of their ‘integration’. Dispensation of integration is not granted to ‘native’ citizens, because those of the so-called ‘second generation’ are born in Western Europe, but they generally do not ‘get’ a dispensation of integration. Neither is it granted to ‘non-immigrants’ (in whatever definition this can have, given the genealogization of integration into ‘n^th^- generation immigrants), because Canadian or US immigrants are generally not included in immigrant integration research reports (and often not even in the research itself), because research focuses on the main ‘problematic groups’. The very calibration of who to include in research is thus crucial, as it of course always is in social science research. In this case, those who are included in research constitute a perfect negative image of who are included in ‘society’.

With a Nietzschean ‘good will to power’, an a priori separation is thus enacted that already does all the work in separating ‘society’ from its ‘immigrants’, but that occurs out of the good will of overcoming – by contributing ‘objective facts’ to the policy issue – precisely that separation. All that can follow are details, numbers, relative differentiations in the ‘distance to society’ that characterize supposed ‘ethnic groups’. These are the things behind the comma, so to speak. What is always already decided, is that *all ethnic groups are at a remove from ‘society’*. People may be well integrated, indeed they may be very well integrated, but that still means they are at the other side of the defining divide – a divide *produced*, no doubt, in a variety of social spheres, but produced in highly specific ways, with the authority of ‘science’ and the legitimacy of ‘objectivity’ and ‘neutrality’, in the social science of immigrant integration. The really decisive difference, after all, is not the difference between the ‘well integrated’ and the ‘less integrated’; it is *the difference between those for whom integration is not an issue at all, and those for whom it is*. To the former, a dispensation of integration applies, and this in effect codes them as ‘society’, and it in turn codes that ‘society’ as ‘white’, precisely by never having to characterize it as such, since ‘whiteness’ is a racial category that is experienced, certainly in the Netherlands, as ‘uneasy’ (Essed & Trienekens, 2008), i.e., as a concept that threatens to undo precisely a whiteness that claims neutrality, non-racial universality.

The specific ways in which immigrant integration is applied to the level of the individual thus in effect purify and immunize a preconceived ‘society’. For as soon as one of a variety of problems occurs – unemployment, incarceration, homophobia – it becomes apparent that these are not problems that exist and occur within society. Rather, these become problems of individuals who (still) reside ‘outside society’ and need to be ‘integrated’. ‘Society’ therefore *has no problems*¸ because any problem there might be is relegated to the individual level of immigrants, who can then be framed group-wise in terms of these problems. This way, ‘society’ is imagined as a pristine, pure domain that is without problems. Problems are problems of ‘integration’, and integration has to do with the position and opinions of non-white individuals. This ‘bringing people into society’ is the rhetoric with which ‘immigrant integration’ is sustained, and it performs the idea that immigrants that have arrived in Western Europe are in fact *still in the process of arriving* (Boersma & Schinkel, 2018). This very presumption of still arriving, of being on the move, in effect renders the children of immigrants into immigrants, which is to say it renders them ‘mobile’, in the sense of somehow still being under way, on their way to ‘society’. The purification performed by ‘immigrant integration’ thus signals the fact that rather than a neutral, theoretical concept (which doesn’t exist anyway), it is a concept of *social hygiene*.

## ‘Immigrant integration’: The mess of measurement

This is built into the individualized measurements of immigrant integration. Taking the Netherlands as a case study, as I do in the book, two broad categories of measures are ‘socio-cultural integration’ and ‘socio-economic integration’. Socio-cultural integration is measured for instance by looking at people’s convictions in terms of ‘secularity’, which is a marker for the degree of ‘modernity’ a person is characterized by. Secularity is then defined – in complete oblivion of the large literature on its meaning, including pivotal works by Asad (2003) and Taylor (2007) – as ‘not churchgoing’ (and ‘church’ includes ‘mosque’ here). The assumption here is – and this in effect functions as a benchmark – that the Netherlands is a ‘secular’ country, and so the more ‘religious’ a person indicates she/he is, the less well ‘integrated’ that person will be. So when approximately a million people in the Netherlands self-identify as ‘Muslim’, researchers do not change their benchmark by concluding ‘maybe the country isn’t that ‘secular’ after all’. Instead, they conclude that there are now up to a million people in need of some degree of further ‘integration’, who thus reside at a certain distance form a ‘society’ that remains, true to the meaning of ‘integration’, an unscathed whole, barely touched by the fact of continuous migration. It becomes clear in this type of measurement that all the work of separation and purification has been done *prior to* the measurement. That is why, in my book, I devote considerable attention to a deconstruction of these measurements.

For something similar occurs in a second way of measuring ‘socio-cultural integration’, which proceeds by way of an inventory of the ‘contacts’ people have with ‘members of other ethnic groups’. Dutch researchers then show how so-called ‘autochthonous’ Dutch have the least contacts with ‘members of other ethnic groups’ (cf. Schinkel, 2013). But they then conclude that some other group, for instance ‘Turks’, actually comprises the least inter-ethnic contacts. This occurs on the one hand because ‘autochthonous Dutch’ are given a dispensation of integration, so their lack of interethnic contacts is of no consequence to them. Moreover, they are also given a dispensation of ethnicity, i.e. they are rendered ethnically ‘neutral’ in a conceptual move that relegates ‘ethnicity’ to all categories of otherness. On the other hand, this occurs because what integration researchers are actually interested in are not ‘interethnic contacts’, but contacts *of* members ‘ethnic minorities’ *with* members of the neutral category, i.e. with ‘autochthonous Dutch’. It then appears that the fact that these ‘native’ Dutch so infrequently meet people from other ethnic groups *is* consequential, *but only for the ‘integration’ of members from these ethnic groups*. Because the two things are of course the same, but not in terms of the conclusions drawn: if ‘native’ or ‘autochthonous’ Dutch have little contacts with ‘members of other ethnic groups’, then it is quite impossible for members of other ethnic groups to have contacts with autochthonous Dutch, because that is exactly the same thing! The point is, here, that these ‘autochthonous Dutch’ are not at all researched as an ‘ethnic group’ whose members can meet members from ‘other ethnic groups’. In fact, members of *all* so-called ‘ethnic groups’ tend to have more interethnic contacts than do ‘autochthonous Dutch’. The point is, rather, that what is actually researched by integration researchers when they deploy the measurement of ‘interethnic contacts’ in this way, is the degree to which ‘members of ethnic groups’ have contacts with ‘autochthonous Dutch’. The latter’s *relative* lack of interethnic contacts are not at issue, because they form the *benchmark* against which ‘other ethnic groups’ (i.e., the only ones actually called ‘ethnic groups’) are assessed. The more contacts with ‘autochthonous Dutch’, the better integrated, but this is not easy, given the fact that one must compete with members of other ethnic groups to have access to the limited number of contacts that ‘autochthonous Dutch’ are able to bring themselves to having with people that are not in their category. Again, as in the measurement of the degree of ‘modernity’, all the work of separating has occurred prior to the actual measurement. The entire set of assumptions, selections, conceptualizations and practices that conditions the work of measuring immigrant integration amounts to a set-up that cannot but operate as a machine for the production of hierarchized differences.

The same goes for the measurement of ‘socio-economic integration’. It is no doubt relevant for emancipatory purposes to study the ways class and race are entangled as expressed by different socio-economic positions. But such questions are exactly what gets obscured by framing the analysis in terms of ‘integration’ and by measuring ‘socio-economic integration’ in terms of the socio-economic positions of immigrants and their descendants relative to the socio-economic positions of ‘autochthonous Dutch’. Why would ‘society’ not comprise the *entire* field of socio-economic statuses? Is it not, rather, that entire field which structures such ‘statuses’ that are only meaningful in a relational perspective to begin with? Why would ‘society’ be defined only by the *mean* of a much larger field of difference and of differentiation? But of course the point is not that society ‘starts’ with a certain socio-economic status or a certain amount of ‘modern’ convictions. ‘Society’ has always already ‘started’ the moment a dispensation of integration is granted to white citizens. And there is no socio-economic status high enough, no cultural assimilation perfect enough, that members of ‘ethnic groups’ can achieve, that qualifies them or their ‘group’ as unproblematically part of ‘society’. They are still in the category of those who need to be monitored, as if they constitute a disease temporarily in retreat, which may resurface at any point – because that is how deep ‘cultural incompatibilities’ go.

What added value does adding this second layer – ‘integration’ – on top of an observed differentiation in socio-economic positions have anyway? In a sense, it has the added value of exorcising particular people, with particular socio-economic positions on a hierarchy that is otherwise uncritically accepted, from the domain of ‘society’. That ‘society’ thus has a benchmark fee in terms of a minimum socio-economic position needed in order to attain entry to it, or so it seems, because ‘society’, in immigrant integration research, does not encompass the *entire differentiation* of socio-economic positions, but it starts from a certain median position. Neither can ‘society’ thus be a dynamic field that *produces* a particular socio-economic differentiation and distribution of capital. In fact, this ‘society’ into which immigrants need to integrate is both everywhere and nowhere. It is everywhere as the benchmark, the domain with which those researched in terms of their ‘integration’ are compared. But it is nowhere, because it is a bloodless, unmovable, undifferentiated, static concept devoid of everything a sociological concept should have. That is why, in measurements of immigrant integration, what people are supposed to be integrated in is never questioned, and is assumed to be constant, and to be entirely unaltered by the presence of those needing to be ‘integrated’ in it. Of course, in the social science of immigrant integration nobody *has a clue* of what the theoretical and conceptual meaning and import of ‘society’ are. In practice, everybody simply uses the notion following common sense’s lack of a need to clarify and make explicit. The result is that if one were to ask for such an explicit conceptual definition of ‘society’, one finds that one step beyond its commonsensical use lies a stunning simplicity, a mushy mesh of exactly the same vagueness and exactly the same unnoticed workings of power as with any commonsensical notion of social life, except this time coming from academics pretending to research people’s ‘integration in society’.

## Imagining ‘society’ against the grain

If this reads like to frontal assault on the concept of immigrant integration, then let me qualify that. Indeed, it *is* a frontal assault on the concept of immigrant integration, but not merely on the concept. The interventions in my book and in this paper are intended also as an assault (of course in the sublimated, academic shape that these interventions assume) on the *practice* of doing research with this concept, on the entire imagination of the social and of ‘social scientific research’ that come with it.

That starts with a theoretical imagination, or lack of it, that conceives of ‘society’ as an entity with an identity, and as an order with a border, in effect positioning social science into the role of border control. Immigrant integration research has, like much social science, always already adopted the organicist genealogy and has espoused an identitarian line of thinking, foregoing theories of difference. The effect of this has been that difference has not been regarded as *constitutive of* the social, but solely as *jeopardizing* it. The only differences that appear in immigrant integration research, are paradoxical *one-sided differences*, i.e., differences exclusively attributed to ‘others’, and taken not as modes of relating and of entanglement, but as signs of problems that are one-sidedly attributed to those considered to introduce these differences from the outside, and into an otherwise unscathed and even unchanged inside. In immigrant integration research, then, ‘society’ has been cast as a whole consisting of parts. And in such a latently organicist conception, historically the concept that has been deployed to mediate between parts and whole is, indeed, ‘integration’. But of course the entire scheme of an encompassing whole consisting of individual parts has been critiqued. It is, in Niklas Luhmann’s terms, a form of ‘old-European thought’. Luhmann offers but one possible alternative to whole/part dualisms, namely a differentiation between social system and environment (see: Luhmann, 1984; Schinkel, 2017). Such an emphasis on *difference* has the advantage to not a priori problematize difference but to consider it as *constitutive* of the social. It has the advantage of not assuming a ‘whole’ to exist, however little observed and researched it is, and to then only look for some coordination among its parts. So it is key to problematize immigrant integration research not only at the surface level of its discourse, but also at the archaeological level of its core epistemic parameters. It then turns out that critiques of immigrant integration research often share such parameters, giving rise to the idea that ‘immigrant integration’ *and* its critiques are actually part of a shared discourse, a discourse characterized by *internal* disputes – a fate that befalls much of what is called ‘critique’. In her recent and otherwise important critique of immigrant integration research, Anna Korteweg for instance argues for renewed attention to “the actual ways in which those labelled ‘immigrants’ are full members of immigrant-receiving societies” (Korteweg, 2017, p. 428). While I agree with much of her critique, the point of my book is to argue that one more effort is needed. We should go one step further and connect the jargon of ‘immigrant integration’ to key issues in social theory. Only then does it become apparent that much of the problematic racialized and gendered routines will recur unless if we stop reproducing the idea that there are ‘societies’ inhabited by ‘members’. For the same reason, it is not enough to ‘de-migranticize migration and integration research’, as Dahinden (2016) has argued. For a start, any claim and practice that concerns ‘integration’ should be *object* of research, rather than the project of research.

In *Imagined Societies*, I likewise state that dualisms of inclusion/exclusion to which critical scholars tend to cling are false to the extent that they fetishize ‘inclusion’ and the ‘inside’, and assume *that ‘exclusion’ is actually possible*. What is possible is rather a differentiation in access to various forms of capital, a differentiation in positions, but such differentiations are – at least in the case of ‘immigrant integration’ – never really ‘exclusions’. A focus on ‘exclusion’ in fact reifies the ‘inside’; into which one can supposedly be ‘included’. A focus on in- and exclusion, sometimes put forward as a critical mode of working for social scientists (Somers, 2008, pp. 136-137), is actually unhelpful because it foregoes the fact that *both inclusion and exclusion are modalities of relating*. In the vocabulary I propose in the book, this means that inclusion and exclusion are two *modes of ‘conclusion’*. A focus on ‘conclusion’ also allows ‘society’ to emerge not as something one can be ‘in’ or ‘out’, but rather as a form of social imagination, i.e. as the fiction of some kind of in- and exclusive sociality, which I call a *confiction* (the fiction of a ‘with’, a togetherness). Confictions such as ‘society’ or ‘immigrant community’ only ever exist through an active work of difference, of separating a supposed ‘inside’ from an ‘outside’, of circumscribing – by means of a work of power-knowledge – who and what is and is not ‘part of society’. Today, the social science of immigrant integration is part of that work of power-knowledge. It may not be the most consequential part, as there are other forms of knowledge production that may have more devastating effects – say, neoclassical or neoliberal economics – but from the perspective of anyone interested in the social science of migration and integration, this is the mirror I wish like to hold up in my book.

If social science wishes to be more than a reiteration, reification and fetishization of confictive self-descriptions, of received common sense or of policy ideologies, it is necessary to imagine ‘society’ against the grain. To imagine what happens when ‘migrants’ move against the grain of today’s ‘immigrant integration’ discourse. It is thus important to undo, or to actively work against, the existing imagination about what happens when people move and settle in another country. The social scientific imagination is so imperiled, that it has become nearly impossible to *not* speak about this process as one in which a ‘host society receives’ migrants that then need to ‘integrate into the host society’. The very historical genesis of notions such as ‘society’ – the very fact that ‘societies’ *have* histories, and highly particular, power-driven ones at that, and moreover that they *will have new histories* – has become completely inaccessible to main stream researchers on immigrant integration. And even if it is accessible, it is practically useless because it cannot in any meaningful way shape research or lay out new pathways of social scientifically imagining the social dynamics of sameness and difference in the context of migration.

Only incremental change is possible, witness the recent turn to ‘super-diversity’, the latest euphemism in concept-work in whiteness that reinvents the wheel and reorders academic hierarchies and CV’s without taking stock of the decades of work in critical race studies, black studies or postcolonial studies. At best, ‘super-diversity’ constitutes an effort to swing the pendulum so far to the other side that all inequalities, all racism and all domination that precedes ‘diversity’ again disappear from view in an effort to let demographics do the work of theory. As Finex Ndhlovu has commented: “superdiversity reinforces the same ideas that it purports to question and challenge – namely, the tendency to homogenize cultural and social groups, and the uncritical embrace of elitist neoliberal conceptualizations of culture and identity” (Ndhlovu, 2015, p. 28). And so one can begin anew to introduce logical inconsistencies and faux-causalities, for instance when the aggregate condition of ‘super-diversity’ is scrutinized for its ‘effects’ (cf. Foner, Duyvendak, & Kasinitz, 2017) in yet another iteration of demographic lumping and splitting. Indeed, the social science of immigrant integration, too, is much more aptly described as a sociology of population groups, a counting and accounting of, and for, ‘population groups’ that it constructs itself, than as a sociology with a social theory. It is a form of applied demographics, and as all demographics, it is part of (bio)political governing (cf. Van Reekum & Duyvendak, 2012; Van Houdt, 2014).

This is indeed exactly what occurs in the Netherlands at present, since ‘super-diversity’ has now been taken on board by the WRR (Wetenschappelijke Raad voor het Regeringsbeleid) [Netherlands Scientific Council for Government Policy], an institution that has left a trail of destruction through the landscape of Dutch migration discourse and policy, having been instrumental in officializing notions such as ‘minorities’ and ‘allochthony’ into policies. By employing ‘super-diversity’ this time around, the WRR aims to merely offer a more fine-grained calibration to the governing of difference under the guise of the accumulation of knowledge or the advancement of insight. It does so by putting forward the idea – preposterous to any postcolonial scholar – that a ‘postcolonial’ perspective (used by the WRR here in a mildly pejorative sense) would mean using distinctions between ‘western’ and ‘non-western’ immigrant groups alongside a few main immigrant groups, whereas a ‘diversity perspective’ entails mentioning all nationalities with which ‘people with a migration background’ are labeled (Jennissen, Engbersen, Bokhorst, & Bovens, 2018, pp. 11-12). Yet all the while, those ‘with a migration background’ are all lumped together over against an apparently primordial ‘native Dutch’ population, thus eclipsing any complexity that might have survived the methodological nationalism of labeling by ‘country of origin’. Each discursive cycle propelled by the WRR is accompanied by the generosity of a combination of good will, critical self-reflection and latest insight – though the insights are never the result of an engagement with the decades of work on racial or postcolonial divisions. The overwhelming continuity is that the presence of ‘others’ – the ones lumped together under the rubric of ‘diversity’, those *who brought diversity with them*, so to say – presents an inherent problem and needs to be governed. Yet only from the ivory tower of power can one keep on reproducing the idea that ‘society’, somewhere, somehow, is something consisting of natives, non-others, something primordially self-similar and merely *confronted* from outside with others, with a ‘diversity’ with which it must now deal. Despite all mobility, there is still a ‘society’ that is not mobile, that can be circumcised out of the super-diverse chaos of movements, trajectories, backgrounds and origins. And in doing so, one never has to reflect on the consequences of the very fact that today’s (post)colonial divisions are the very effects of these ‘societies’ being on the move, enacting violent histories of continuing movement and influence – histories that expose the entire idea of ‘people with a migration background’ as the governing fictions they are. ‘Super-diversity’, in this sense, constitutes the continuation of immigrant integration by other means. And one must hasten to add: the means may differ, but the institutions, the flows of money and the academic CV’s hardly do so.

## The colonial archive: Social science and/as methodological whiteness

The question is, can we come to concepts and tools in which to explain social science to ourselves, for reasons beyond the fact that the current modes are simplistic, anti-intellectual, untheoretical and self-contradictory? If I can venture a guess, and if in doing so I am permitted to be optimistic, I would say that the sociology of immigrant integration stands a good chance of one day being judged as a theoretical hiccup of a still young discipline, as one of those paths, like social Darwinism, into which researchers once strayed and made careers in meaningless imitations of normal science, a historical oddity that did nothing to further either the complexity with which the discipline grasps the social world or the public knowledge which helps publics and collectives gain insight into themselves. But of course to say that this research will one day be looked upon as a mere fluke, an ‘oddity’, is to say too little, and it is a too depoliticized judgment. Rather, this oddity will likely be placed under the historically recurring, or even continuous, brand of practices that can rightfully be called *neocolonial*. Without undue social scientific narcissism about the importance of what we do, this is where things get to be consequential.

The social science of immigrant integration is a typically ‘modern’ endeavour, in the sense that it assumes the position of societal judge. Much of early sociology (and as Foucault has shown (1999), the same goes for much of psychology) was likewise about assessing the degree to which persons could be judged ‘modern’ over against ‘traditional’, ‘normal’ over against ‘pathological’, ‘civilized’ over against ‘degenerate’. It posited the existence of ‘society’ not merely in a descriptive way, but by way of marking the domain of the already-emancipated, the already-elevated to the status of civilized, advanced, developed, progressed. Much of sociology then became concerned either with the formal structures that characterized such societies (division of labour, bureaucracy), or with those people who were yet to become a part of them. If much of social science in general has been interested in the unassimilated, the deviant or the abnormal as a way of learning about what was assimilated and normal, it did so largely by never questioning the validity of such descriptions, thereby indeed contributing to the taken-for-grantedness that these descriptions wielded. In this vein, social science has been a practice of record keeping, of keeping score on who could be considered an unproblematic member of society, and why was to be labeled as not – not yet – conforming to it, i.e., as remaining in non-modern conditions. Of course, this record keeping has been enormously effective in producing the pathologies it claimed to objectively describe. Many early sociologists explicitly wrote about their vocation in terms analogous to, or directly borrowed from, medical science and practice. Durkheim’s idea of a ‘therapeutic sociology’ is a notable example, but more generally Wright Mills’ description of ‘social pathologists’ has been apt (Wright Mills, 1969). On the use of concepts such as ‘society’ or ‘American culture’, Wright Mills said:

“The terms represent undifferential entities. Whatever they may indicate, it is systematically homogeneous. Uncritical use of such a term as “the” permits a writer the hidden assumption in politically crucial contexts of a homogeneous and harmonious whole. The large texture of “the society” will take care of itself, it is somehow and in the long run harmonious, it has a “strain toward consistency” running through it; or, if not this, then only cooperation of all is needed, or perhaps even a right moral feeling is taken as a solution” (Wright Mills, 1969, p. 538).

In much of the social scientific work on immigrant integration, much the same is expected of the ‘right moral feeling’.

This kind of social scientist as record keeper has been concerned with ‘assimilation’ at home, but (s)he had first of all practiced this abroad, in colonial contexts. And the colonial situation of moral monitoring is a key moment in the genealogy of the study of immigrant integration. Dutch social scientists of immigrant integration, for instance, have as their predecessors those who assessed the morals of Europeans of mixed blood and of ‘poor whites’ in the East Indies (Stoler, 2002, 2009). Stoler refers to ‘moral measurements’ to denote the ways in which administrators in the nineteenth century tried to assess the “complete suitability for European society” which was a condition for the granting of citizenship rights (Stoler, 2002, p. 17). In a similar vein, Gouda has noted that the Dutch colonial regime rendered different populations manageable by placing them in a taxonomy of ‘ethnic traditions’ based on European assessments of cultural taste and refinement (Gouda, 1995). As Hondius has shown, for many people outside Europe, migration to Europe became feasible only for a brief period following the end of formal colonialism. That period was brief because the end of the colonial era soon meant restrictive immigration policies for formerly colonialized and enslaved peoples (Hondius, 2011). To that we may add that the ones who did migrate were met with the setting up of an increasingly elaborate system of monitoring and record keeping that reproduces their otherness, and hybridizes it because it mixes various groups under the sign of ‘integration’. Whereas Hondius (2011) shows that for centuries, laborious efforts were undertaken to keep enslaved people and slavery itself out of sight from native Dutch, once present in the Netherlands this monitoring system – which is in all respects an apparatus of *moral monitoring* – was set up to *maximize the visibility of otherness*.

Just to be clear, then: yes, measuring immigrant integration is a thoroughly neocolonial practice. It comes out of a history in which the encounter with the other first emerged, and emerged by way of a raced work of cultural classification and in the context of dominance. It is a form of what Ann Stoler has recently called ‘colonial duress’: the tenacious survival of colonial effects and divisions (Stoler, 2016). And it exists today in contexts of power asymmetries that in turn help shape the raced classifications and ethnic taxonomies of researchers. The very whiteness of the research community is a case in point here, and it has been noted by scholars working in the tradition of critical race studies (cf. Essed & Nimako, 2006). The prevailing attitude among white researchers is that their whiteness should not and does not matter in their research. Theirs, in other words, is a ‘white innocence’, in terms of Gloria Wekker (2016). And this is not a surprising denial, given the overwhelming whiteness of the field, the one-sidedness oif the curricula that shape the minds of new members of the field, and the reward systems in place which prioritize sameness and exclude otherness within academia and governmental research institutes. I would say that I myself had, in a way, to be re-educated in order to become sensitive to the effects of whiteness, and this re-education is by no means concluded. One can *instinctively* deny, as most researchers do, that one’s whiteness matters, but instinctive responses hardly qualify as scholarly considerations. And indeed, the literature on whiteness is generally not considered in the main stream of research on immigrant integration. Which is why ‘whiteness’ is still often naively taken to refer to ‘skin color’, whereas it pertains first and foremost to a system of *domination* that is reproduced by the off-hand, instinctive denial of its very existence.

Whiteness is what gives credence to the methodological nationalism that provides privileged New York scholars such as Alba and Foner (2015) with the tools to evaluate large parts of the world in terms of people’s ‘integration’ – and the arrogance of privilege clearly shines through in Alba and Foner’s response (2016) to Adrian Favell’s nuanced critique of their book (Favell, 2016). And methodological nationalism in effect also means what Gurminder Bhambra has recently called *methodological whiteness* (Bhambra, 2017). Methodological whiteness has real consequences. Structural and institutional racism are not unrelated to direct, personal forms of racism, if only in terms of convictions. Decades of affirmations donned in the authority of ‘science’ that migrant others are ‘lagging behind’, that they are here but not in an ‘integrated’ way, ultimately add plausibility to the racist inference that ‘indeed, if they take this long to fully arrive here, to be well integrated, they’re probably essentially unfit to do so’. And this is what is made quite explicitly in conceptions of incompatibility between ‘the West’ and ‘Islam’ or ‘modernity’ and ‘migrants’ – and the racism starts, of course, with the very setting up of such oppositions. In other words, the artificial separations produced by scholarly work on immigrant integration always already are political in the ways in which the may (re)affirm racist conceptions either out of fatalism or out of hatred. The lags and gaps that become visible and available for political action only *after* they have been conjured in academic research based on childishly simplistic assumptions and a bewildering lack of reflexivity are in no way innocent renditions of ‘realities out there’. Political pundits and academic bureaucrats alike are fond of siding with such naïve realism and descry that ‘postmodernism denies realities’, and with dire political consequences because the unequal situations people are in go unrecognized. But in fact one needs to move beyond such affirmations of commonsense realities and recognize that one can only have a ‘realist’ perspective on social reality if one accounts for its complexities, that is, if one takes social reality *more seriously* than tends to be done in research that spits out some numbers on ‘society’ and its ‘non-integrated’. These complexities pertain both to the entanglement of the social with the social sciences – the fact that the social sciences are not in any way external to their research object – and to the ways in which regional, national notions of ‘societies’ consisting of individual members in no way do justice to the realities of local *or* global entanglement so poorly described by positing fixed ‘societies’ versus ‘migrants’.

That means these complexities also pertain to the historical ways in which capital and race are globally intertwined to produce excess populations, cheap labor, and human expulsions. The point is, then, that a social science interested in what happens to migrants when they move, and to what happens in the larger social ecology of their movement, should move beyond the confines of the confictions (‘national societies’) to which it clings and in which it *believes*. Rather, it should pay due attention to the enduring consequences of the fact that, as Cedric Robinson already said in 1983, “there has never been a moment in modern European history (if before) that migratory and/or immigrant labor was not a significant aspect of European economies. That this is not more widely understood seems to be a consequence of conceptualization and analysis: the mistaken use of the *nation* as a social, historical, and economic category; a resultant and persistent reference to national labor ‘pools’ (e.g., ‘the English working class’); and a subsequent failure of historical investigation” (Robinson, 1983, p. 23).

## Conclusion: For a social science against immigrant integration policy

As social scientists, who are our publics? This is a question that, in concluding, I propose social scientists should ask themselves more often. The question is not ‘who are our research funders?’, though research funders are obviously one type of public. Too often, the intricate ties between social scientists and policy makers – both financially and conceptually – seem to hinder progress in understanding migration processes. And yet there is, and has been, lots of research not centering on ‘integration’. In a variety of disciplines, and in fact often interdisciplinary in scope, researchers have scrutinized migration and its consequences in ways that move beyond ‘integration research’. Vollebergh analyzed living together in Antwerp based on a fine-grained, in-depth ethnographic study (Vollebergh, 2016). Van Houdt (2014) has changed the conversation about ‘immigrant integration’ by subjecting it, for the first time, to an analysis in the Foucaultian framework of technologies of government. Van Reekum (2014) has meticulously traced the interconnections between the rise of ‘immigrant integration’ and the transformations of Dutch nationalism. Francio Guadeloupe has deconstructed the racial formation that governs the Dutch-Carribean (post)colonial relations to show how there could be no ‘Dutch nation’ if it is not also what he experiences as ‘a Carribbean island’ (Guadeloupe, 2013). Mepschen (2016) has turned the tables in an extended ethnography of ‘everyday autochthony’ in light of a ‘culturalization of citizenship’. And Çankaya has illustrated how ethnic and racial categories intricately tied up with integration discourse do everyday work in surveillance and policing institutions (cf. Gowricharn & Çankaya, 2015). These are but a few examples of a new generation of researchers waeving together perspectives from sociology, anthropology, critical race studies, postcolonial studies and feminist theory. Immigrant integration research, while purportedly also talking about the realities of migration, has been ignorant about this kind of work, which doesn’t fit with its approach of using large-scale longitudinal surveys. Over against detailed ethnographic sensibility, such detachment and abstraction is what passes for ‘objectivity’ for those wedded to bureacuratic categories and state money.

Strangely enough, in many of the encounters I have had with social scientists studying immigrant integration, the kind of critical perspective I espouse here has been called both ‘normative’ and ‘non-objective’, but at the same time it has been called ‘unrealistic’, ‘non-applicable’ and not ‘policy relevant’. Much could be said about such qualifications by way of the implicit philosophy of science from which they stem (a philosophy of science about a hundred years old), but here I merely wish to highlight the paradox such notions entail. Because a critical analysis is deemed *both* unduly normative *and* non-applicable as in not useful for policy. Whereas the point I’m making may well be phrased thus: conventional analyses of immigrant integration are *too little ‘objective’* because they have *too little distance* vis-à-vis the categories, questions and problematizations that prevail in public discourse and in policy contexts. In this sense, an ‘objective’ account of the phenomenon ‘immigrant integration’ would be one that asks: what happens when some people are evaluated in terms of their ‘integration’ and others are not? It would not a priori accept the particular meaning the natives attach to the term ‘immigrant integration’, but it would study what happens when they give it the meaning that they do. Either way, I think the ‘objectivity’ of an analysis is tied to the way in which an account of its situatedness is provided (Haraway, 1991), but if research want to speak of ‘objectivity’ by way of the ‘mechanical objectivity’ that Daston and Galison discuss, i.e., by way of an effort at excluding the interference of the researcher in the analysis, then it would be fitting to note that an uncritical acceptance of all the key concepts of the analysis, taken from public discourse and policy programs, would not qualify as scientifically ‘objective’.

Again, I’m paying attention to this issue not to bicker over whose perspective is truly ‘objective’, but merely because it makes a point about the schizophrenic situation from which much work on migration that considers itself to be ‘non-critical’ actually emerges. It claims *both* more neutrality *and* more applicability in highly normative political contexts. And this is not merely a Weberian way of claiming that political contexts need to make decisions based on independently produced, objective knowledge. Because the genealogy of research on immigrant integration shows precisely this: that the categories, questions and modes of analysis of social science cannot be separated from those of the state. In fact, much immigrant integration research in Western Europe comes out of particular entanglements between university-based social scientists and state institutions. Often, state institutions set – and finance – surveys that others also work with, as is the case in the Netherlands with the Netherlands Institute for Social Research or in Germany with the SOEP survey. Categories are shaped and reshaped in research communities that run laterally across academic and policy fields (Boersma, 2019). This type of entanglement is nothing special; it is in fact central to much of (social) science, as has been argued in work on the ‘social life of methods’ (Law, Ruppert & Savage, 2011).

This entanglement is nothing special, indeed, but it is highly consequential, and we live in an era where we cannot afford to simply reproduce dominant divisions. In a time of resurgent racisms, of widespread populist resentment against fictive ‘mass migrations’, a time when political leaders across Europe and in the United States increasingly take explicit racism on board, but also in which migration policies deemed ‘decent’ and by now thoroughly conventional in fact constitute a deliberate politics of death (think of the Mediterranean Sea), social scientists are forced to take sides. We can no longer afford to claim either neutrality or policy-relevance in the interests of the migrant others whose scores they keep. Anyone now working on migration and immigrant integration should know that whatever choices (s)he makes are on record and constitute a historical archive that will be judged not on its brilliant scientific finds (who’s banking on that?!), but on the extent to which it contributed to forms of knowledge that could contribute to public discourses enabling forms of life outside the registers of an overwhelmingly white dominance. In this sense, our research choices, which co-shape public discourse and are closely entangled with state policies, are always already political. And it is no longer a question of being just a little racist, of insisting just a little bit that those who ‘were here before’ get to set the agenda for those who came later. The agenda of those who insist on ‘immigrant integration’, and who thereby a priori assume that migrants have not really arrived, are not yet ‘members of society’ is in its effects only slightly removed from the explicit racism of the current white backlash on the (alt-)right.

This is the urgency of the issue. It does not concern the mere measuring of achievements and positions. It is not limited to the dull findings we present at meetings where we all suffer from bureaucratic boredom. Its effects are not exhaustively subsumed under the greyness of our conference halls. Its workings are not limited to the pages, papers and CV’s we produce off of it. The question is ultimately whether we want to make resources available for racist modes of relating to migrants and their children, or for alternatives.

## Abbreviation

WRR: Wetenschappelijke Raad voor het Regeringsbeleid [Netherlands Scientific Council for Government Policy]
